# Clinical practice guideline protocol for perioperative glycemic control in diabetic and nondiabetic adults undergoing noncardiac surgery

**DOI:** 10.1002/gin2.70019

**Published:** 2025-04-05

**Authors:** Fabricio Andres Lasso Andrade, Nubia Fernanda Sanchez Bello, Jose Hugo Arias Botero, Jaddy Sandrey Bedoya, Luz Maria Gomez Buitrago, Alexandra Chaves Vega, Fernando Ríos Barbosa

**Affiliations:** ^1^ Universidad Nacional de Colombia Bogotá Colombia; ^2^ Fellowship en Dolor y Cuidado Paliativo Universidad CES, Facultad de Medicina Medellín Colombia; ^3^ Facultad de Medicina Universidad CES Medellín Colombia; ^4^ Sociedad Colombiana de Anestesiología y Reanimación (S.C.A.R.E.) Bogotá Colombia; ^5^ Universidad de Caldas Manizales Colombia; ^6^ Universidad de La Sabana, Facultad de Medicina Chía Colombia

**Keywords:** CPG, glycemic control, noncardiac surgery, perioperative, Type II diabetes mellitus

## Abstract

**Introduction:**

Type II diabetes mellitus (DM II) is a chronic and prevalent disease affecting millions of people worldwide, with a significant impact on public health. This protocol outlines the methodology for developing a Clinical Practice Guideline (CPG) focused on managing glycemic control in diabetic and nondiabetic adults undergoing noncardiac surgery. The objective of this guideline is to provide recommendations based on the best available evidence, improving the quality of care and clinical outcomes for these patients.

**Methods:**

The CPG will be developed using the GRADE methodology, a rigorous and transparent approach that allows for the evaluation of evidence quality and the formulation of robust recommendations. The process will include identifying critical clinical questions using the PECOT format, ensuring a comprehensive evaluation of perioperative glycemic management.

**Questions:**

The clinical questions addressed in this CPG cover key aspects of perioperative glycemic management, from defining preoperative hyperglycemia to strategies for intraoperative and postoperative monitoring. These questions were prioritized through a modified Delphi process, ensuring their clinical relevance.

## Highlights

1. Perioperative hyperglycemia increases the risk of surgical complications.
  - Pre‐, intra‐, and postoperative hyperglycemia has been associated with higher rates of infections, reoperations, prolonged hospital stays, and mortality in surgical patients.
2. The guideline follows the GRADE methodology to ensure high‐quality evidence.
  - The Clinical Practice Guideline (CPG) was developed using the GRADE approach to evaluate evidence quality and generate strong, evidence‐based recommendations.
3. Clinical questions were prioritized using a modified Delphi method.
  - From an initial list of 45 questions, 19 were selected through a consensus process, with 11 ultimately prioritized for recommendation development.
4. The protocol considers disparities in access to technology and treatments in Colombia.
  - Unlike international guidelines, this CPG adapts recommendations to Colombia's resource availability, ensuring its applicability in different clinical settings.
5. The guideline defines clear criteria for perioperative glycemic management.
  - It includes specific preoperative glucose thresholds that may contraindicate elective surgery, intraoperative monitoring strategies, and insulin management recommendations for the postoperative period.
6. The aim is to reduce variability in clinical practice.
  - The CPG standardizes glycemic control approaches to improve patient safety and optimize surgical outcomes, promoting evidence‐based care.
7. The document has undergone external review by anesthesiology experts.
  - The Colombian Society of Anesthesiology and Resuscitation (SCARE) reviewed and validated the recommendations to ensure their accuracy and clinical applicability.
8. The protocol has been officially registered for transparency and methodological rigor.
  - It is registered in the PREPARE platform under PREPARE‐2024CN524, ensuring compliance with international standards for clinical guideline development.

## 1 INTRODUCTION

Type II diabetes mellitus (DM II) is a chronic condition characterized by hyperglycemia due to insulin resistance or insufficient secretion. Globally, it affects 537 million people, with an estimated prevalence of 10.5% in adults.^1^ In South America, countries like Colombia estimate that up to 25% of diabetes cases remain undiagnosed,^2^ further complicating optimal glycemic management in the perioperative context. In Colombia, DM II affects 3.3 million individuals, with a prevalence of 7.4% in men and 8.7% in women.^3^

DM II is associated with various complications, including increased risks of cardiovascular,^4^ neuropathic,^5^ renal,^6^ and ophthalmologic^7^, ^8^ issues. These complications are attributed to poor glycemic control over the course of the disease.^4^ Studies have demonstrated that poor glycemic control in surgical patients is linked to higher rates of infections, reoperations, prolonged hospital stays, and mortality.^9^, ^10^

De novo perioperative hyperglycemia has also been associated with long‐term adverse outcomes. Patients with de novo hyperglycemia have higher mortality rates (16% vs. 3%, *p* < 0.01) compared to those with diabetes or normoglycemia (16% vs. 1.7%, *p* < 0.01).^11^ A retrospective study of Noncardiac surgery patients found that those with stress hyperglycemia (glucose 111–140 mg/dL) had a higher incidence of surgical site infections (OR 3.61, 95% CI 1.22–10.67), and glucose levels above 220 mg/dL increased the postoperative infection risk to OR 12.13 (95% CI 3.71–39.64).^9^

Although international guidelines exist, they have been developed in contexts different from Colombia, with predominantly Caucasian populations and access to technological and pharmacological resources often unavailable in middle‐ and low‐income countries like Colombia.^12^, ^13^ These guidelines do not address specific limitations in access to healthcare services or differences in the prevalence of perioperative complications related to glycemic control in non‐Caucasian patients. In Colombia, unequal access to continuous glucose monitoring technology and limited availability of standardized protocols represent significant barriers to implementing international recommendations.^14^ Furthermore, the variability in glycemic control targets established by international diabetes associations underscores the need to define specific ranges for perioperative management in populations with demographic and epidemiological characteristics distinct from Caucasians, taking into account the resources available in Colombia.^15^ Current evidence shows variability in perioperative glycemic control ranges, creating confusion among clinicians and potentially leading to adverse outcomes due to inconsistent management based on the differing resources in middle‐ and low‐income countries.

Developing a Clinical Practice Guideline (CPG) for Colombia on glycemic control management in diabetic and nondiabetic adults undergoing noncardiac surgery would improve standardized care and healthcare quality to reduce complications and enhance clinical outcomes.^16^ The recommendations would provide guidance to reduce variability in management, promote evidence‐based practices, and improve patient‐centered care.

### 1.1 Scope of the CPG

The scope of this CPG, including the included questions, was developed using the PECOT model, which encompasses the patient population (P), the exposure or intervention of interest (E), the comparator when applicable (C), the desired or evaluated outcomes (O), and the timeframe (T) in which outcomes will be observed—in this case, the perioperative period.

The PECOT model was chosen for this CPG due to its ability to incorporate both intervention‐based and exposure‐based questions, allowing for a more comprehensive evaluation of perioperative glycemic management.^17^ This is particularly relevant in the context of type II diabetes mellitus and glycemic control in patients undergoing noncardiac surgery, where not all questions involve direct comparisons of interventions but instead observe outcomes over time.

### 1.2 Objectives

The CPG aims to establish clear, evidence‐based standards for glycemic management during the perioperative period. It seeks to identify patients at risk of complications related to hyperglycemia and hypoglycemia, provide recommendations for preoperative management and intraoperative and postoperative monitoring of glycemia, and offer interventions to reduce perioperative complications. Additionally, it focuses on enhancing anesthesiologists' competencies in glycemic management to optimize surgical outcomes and minimize postoperative complications.

## 2 GUIDING QUESTIONS AND OUTCOMES

The Guideline Development Group identified and prioritized 11 questions (Supporting Information: Table 1). These questions were selected through a process based on the modified Delphi method.^18^ Initially, 45 potential questions were identified, followed by three selection phases that narrowed them down to 19, and finally prioritized to 11 based on clinical relevance, variability in clinical practice, and clinical significance, using a voting scale of 1 to 9 according to the modified Delphi method. The questions address glycemic management in the perioperative period, including patients with hyperglycemia and hypoglycemia across all perioperative phases.

### 2.1 Patient population

Patients with diabetes mellitus, de novo hyperglycemia, or at risk of hyperglycemia or hypoglycemia undergoing noncardiac surgery.

### 2.2 Exposure

The exposures of interest in this guideline include specific preoperative glycemic levels that may influence decisions to postpone or cancel elective surgical procedures. Additionally, the criteria and definitions for diagnosing preoperative hyperglycemia, as well as HbA1C measurement during preanesthetic evaluations, will be considered. Perioperative management strategies for patients using insulin pumps or receiving insulin or hypoglycemic medications will also be addressed, alongside identifying risk factors for hyperglycemia and hypoglycemia in the preoperative period.

### 2.3 Comparator

The comparators in this guideline will include different glycemic thresholds or the absence of a defined threshold to guide clinical decisions. Alternative definitions of hyperglycemia and their management effectiveness compared to standard care will also be evaluated.

### 2.4 Outcomes

The expected outcomes of the proposed interventions and strategies include improved clinical decision‐making, particularly regarding the postponement or cancellation of elective surgeries based on glycemic levels. The guideline also aims to optimize risk assessment and preoperative management, prevent and control glycemic alterations, reduce postoperative complications, and improve surgical and perioperative outcomes.

### 2.5 Timeframe

The timeframe encompasses the preoperative, intraoperative, and postoperative phases, considering evaluation, management, and follow‐up of glycemic control at each stage to maximize clinical outcomes and reduce associated complications.

## 3 METHODOLOGY

The development process of this Clinical Practice Guideline (CPG) will be conducted using a de novo methodology based on the GRADE (Grading of Recommendations Assessment, Development, and Evaluation) framework.^19^ This formal approach to creating CPGs focuses on developing new and specific recommendations for our area of interest, ensuring that they are grounded in the best available evidence and tailored to the local context and patients' needs.

The GRADE methodology was chosen to guide the evaluation of evidence quality and the strength of recommendations. This system allows for a comprehensive and transparent assessment of scientific evidence, facilitating recommendations that reflect both the quality of the evidence and the balance between benefits and risks.

The development process of this CPG will include the following key phases:

**Configuration:** Identification and selection of relevant clinical questions, formulated using the PECOT format to ensure a comprehensive approach that includes exposure, comparison, and relevant outcomes.

**Evidence Assessment:** Systematic literature review to identify and evaluate the quality of evidence related to the formulated questions. The GRADE methodology will be applied to rate the quality of evidence and develop recommendations based on the findings.

**Formulation of Recommendations:** Recommendations will be collaboratively developed by a multidisciplinary panel of experts using the GRADE methodology to ensure they reflect available evidence and patient preferences and values.

**External Review and Quality Assurance:** Preliminary recommendations will undergo an external review by experts from the Colombian Society of Anesthesiology and Resuscitation (SCARE) to ensure relevance and applicability in the clinical context.

**Finalization and Reporting:** The final CPG report will be prepared following the criteria established by the AGREE (Appraisal of Guidelines for Research & Evaluation) tool,^20^ ensuring the guideline meets standards of rigor, clarity, and applicability.

The final report will be divided into sections that include (I) basic information; (II) scope; (III) rigor of development; (IV) recommendations; (V) external review and quality assurance; (VI) funding, declaration, and management of conflicts of interest; and (VII) other relevant information.

### 3.1 Protocol registration

This Clinical Practice Guideline (CPG) protocol has been registered on the ‘PREPARE’ platform (Practice Guideline Registry for Transparency) under the registration number PREPARE‐2024CN524, with the registration date of June 28, 2024. This platform is managed by the World Health Organization Collaborating Centre for Guideline Implementation, Dissemination, and Knowledge Translation at Lanzhou University's Evidence‐Based Medicine Centre.

### 3.2 Guideline development group

The GDG will consist of a multidisciplinary team of experts in clinical research and medical practice, with a particular focus on the perioperative management of patients with metabolic disorders. This group will include clinical professionals with expertise in anesthesiology, who will provide practical knowledge and evidence‐based medicine competence for developing the CPG.

The group leader, a physician with extensive clinical research experience, will efficiently coordinate meetings, establish clear protocols, and foster a collaborative environment among all members. The methodological expert, an anesthesiologist and epidemiologist specializing in evidence evaluation and clinical guideline methodology, will be responsible for formulating clinical questions and leading the evidence evaluation process. Clinical professionals, all anesthesiologists experienced in perioperative glycemic control, will contribute practical knowledge and review relevant studies to assist in formulating recommendations.

Before starting this guideline, all group members signed a conflict‐of‐interest declaration to ensure process transparency. If new members join the development group, they will be informed of the standards established from the beginning and required to comply with them.

### 3.3 Searches and evidence assessment

The literature searches will be conducted exhaustively and systematically using databases such as PubMed, Google Scholar, Embase, and BVS‐LILACS. The goal is to identify available evidence related to the clinical questions formulated for the development of the CPG.

The evidence assessment will follow a tiered approach, starting with identifying systematic literature reviews (SLRs). If relevant SLRs are unavailable, clinical trials will be sought, and observational studies will be considered if higher‐level evidence is unavailable.

To evaluate the methodological quality of systematic reviews,^21^ clinical trials,^22^ cohort studies, case‐control studies, and cross‐sectional studies, the JBI critical appraisal tool^23^ will be used. This tool allows for rigorous and standardized assessment, applying specific checklists for each study type to address potential biases and maintain consistency in evidence evaluation throughout the guideline development process.

### 3.4 Evidence and eligibility criteria

The development group will be responsible for building the evidence base for the CPG on glycemic control in diabetic and nondiabetic patients undergoing noncardiac surgery. This process will focus on identifying, evaluating, and synthesizing systematic reviews, clinical trials, and observational studies, in that order of priority, to support the recommendations.

The primary evidence source considered will be systematic literature reviews (SLRs). These reviews must have been developed using rigorous methodologies, preferably with the GRADE system, and accompanied by a clear and detailed report on the methodology used. If relevant or acceptable‐quality systematic reviews are unavailable, clinical trials will be identified and evaluated. These studies must provide direct evidence for the clinical questions formulated in the CPG and meet high methodological standards. In the absence of suitable systematic reviews or clinical trials, observational studies will be considered. Although these studies offer a lower level of evidence, they can be valuable in the absence of higher‐quality research and will be carefully evaluated for relevance and methodological rigor.

Case reports, case series, and studies with significant methodological deficiencies that may compromise the validity of the results will be excluded.

The quality of identified evidence will be assessed using the SIGN tool, which classifies evidence into high (++), acceptable (+), or unacceptable (−) quality. Only studies classified as high or acceptable quality will be used to formulate recommendations.

### 3.5 Guideline reporting

#### 3.5.1 The AGREE instrument

The AGREE (Appraisal of Guidelines for Research & Evaluation) instrument will be used as a tool to structure and report the CPG development. AGREE provides a robust framework to ensure that the CPG is reported clearly, transparently, and with adequate methodological rigor, covering all necessary aspects for its implementation and use in clinical practice.^24^

### 3.6 Evidence quality and recommendation strength

Evidence quality and recommendation strength will be evaluated using the GRADE approach. This method ensures a clear and rigorous assessment, facilitating clinical decisions based on the best available evidence.

Evidence quality will be classified into four levels: high, moderate, low, and very low. These levels reflect confidence that the estimated effect observed is close to the true effect. High‐quality evidence implies great confidence that the true effect is close to the estimate, while very low‐quality evidence indicates a high likelihood that the true effect substantially differs from the estimate.

Recommendation strength will be categorized as strong or weak, depending on the GDG's confidence that the benefits of an intervention outweigh its undesirable effects, or vice versa. A strong recommendation suggests that benefits clearly outweigh risks, and most are expected to follow this guidance. A weak recommendation indicates that benefits probably outweigh risks, but individual preferences or specific contexts may lead to differing decisions.

### 3.7 External review

The external review will focus on clinical content. This task will be carried out by expert anesthesiologists from the Colombian Society of Anesthesiology and Resuscitation (SCARE), who will evaluate the accuracy and relevance of the recommendations.

### 3.8 Conflict of interest management

All collaborators of this CPG protocol declared their conflicts of interest upon joining the guideline development group. The report was reviewed by the methodological advisor and group leader, and no conflicts of interest were found that could affect or bias any author's evaluation or recommendations.

## AUTHOR CONTRIBUTIONS

Conceptualization, methodology, and supervision: Fabricio Andres Lasso Andrade, Nubia Sanchez, and Jose Hugo Arias. Drafting the manuscript: Fabricio Andres Lasso Andrade. Review and editing: Nubia Sanchez, Jose Hugo Arias, Fernando Rios, Luz Maria Gomez Buitrago, and Alexandra Chaves (members of the guideline development group). All authors reviewed and approved the final version of the manuscript.

## CONFLICT OF INTEREST STATEMENT

The authors declare no conflict of interest.

## ETHICS STATEMENT

The authors have nothing to report.

## Supporting information

Supporting information.

Supporting information.

## Data Availability

The data that support the findings of this study are available from the corresponding author upon reasonable request. The data supporting the guideline protocol are available in the tables, figures, and supplementary files attached to this article. For additional information, interested parties may contact the corresponding author with a reasonable request.
