# Supplementary material for: Clinical practice guideline protocol for perioperative glycemic control in diabetic and nondiabetic adults undergoing noncardiac surgery
Source: Clin Public Health Guidel. 2025 Apr 5;2(2):e70019. doi: 10.1002/gin2.70019 (PMC13563344; doi:10.1002/gin2.70019)
Supplement: Supplementary file 1 [file GIN2-2-e70019-s002.docx]

**Table 1. Clinical Questions.**

| **Question** | **Expected Outcome** | **Objective of the Guideline** |
| --- | --- | --- |
| **How is hyperglycemia defined in the preoperative period?** | Establish a clear and specific definition of hyperglycemia in the preoperative context. | Define Standards for Perioperative Glycemic Control |
| **What is the preoperative glucose level at which a patient should not undergo elective non-cardiac surgery?** | Determine a glucose threshold that contraindicates an elective procedure to prevent complications. | Identify Patients at Risk |
| **In which patients should HbA1C measurement be performed during the pre-anesthetic evaluation?** | Identify patients who would benefit from HbA1C measurement to optimize glycemic control. | Identify Patients at Risk |
| **What are the risk factors to consider when measuring preoperative glucose for the risk of hyperglycemia or hypoglycemia?** | Provide a list of risk factors that justify glucose measurement in the preoperative period. | Identify Patients at Risk |
| **What is the preoperative management of hypoglycemic medications in patients who are receiving them?** | Provide recommendations on adjustments to oral medication and insulin in the preoperative period. | Perioperative Management of Glycemia in Non-Cardiac Surgeries |
| **What are the target glucose levels to maintain during the intraoperative period?** | Establish target glucose ranges to maintain during the intraoperative period to prevent complications. | Provide Monitoring Strategies |
| **What are the insulin or dextrose management protocols and glucose monitoring strategies that prevent postoperative complications in patients with hyperglycemia or hypoglycemia?** | Define effective protocols for insulin and dextrose management, as well as glucose monitoring in the postoperative period. | Reduce Perioperative Complications |
| **How should perioperative management of patients using insulin pumps be conducted?** | Establish clear guidelines for managing insulin pumps during the perioperative period. | Perioperative Management of Glycemia in Non-Cardiac Surgeries |
| **What is the impact of preoperative, intraoperative, and postoperative hyperglycemia on the incidence of postoperative complications?** | Determine the relationship between hyperglycemia and postoperative complications to enable preventive interventions. | Reduce Perioperative Complications through Optimized Glycemic Control |
| **How should insulin infusion be managed in the postoperative period?** | Provide a clear protocol for insulin infusion management in the postoperative period to ensure proper glycemic control. | Reduce Perioperative Complications |
| **What is the appropriate glucose monitoring protocol in the postoperative period for both diabetic and non-diabetic patients?** | Detail an appropriate postoperative glucose monitoring protocol to ensure proper glycemic control in all patients. | Provide Monitoring Strategies |
